# Extracellular Adenosine in Gastric Cancer: The Role of GCSCs

**DOI:** 10.3390/ijms26157594

**Published:** 2025-08-06

**Authors:** Sharin Valdivia, Carolina Añazco, Camila Riquelme, María Constanza Carrasco, Andrés Alarcón, Sebastián Alarcón

**Affiliations:** 1 Cancer Biology Laboratory, Facultad de Medicina, Universidad San Sebastián, Sede Concepción, Campus Tres Pascualas, Concepción 4080871, Chile; sharin.valdivia@uss.cl (S.V.); alarcon.andres1707@gmail.com (A.A.); 2 Departamento de Ciencias Biológicas y Químicas, Facultad de Ciencias, Universidad San Sebastián, Sede Concepción, Campus Tres Pascualas, Concepción 4080871, Chile; 3 Nutritional Biochemistry Laboratory, School of Nutrition and Dietetics, Faculty of Rehabilitation and Quality of Life Sciences, San Sebastian University, Valdivia 5091000, Chile; carolina.anazco@uss.cl; 4 Escuela de Medicina, Facultad de Medicina, Universidad San Sebastián, Sede Concepción, Campus Tres Pascualas, Concepción 4080871, Chile; camila.riquelme@uss.cl (C.R.); constanza.carrasco@uss.cl (M.C.C.)

**Keywords:** adenosine, stomach cancer, gastric cancer, gastric cancer stem-like cells (GCSCs)

## Abstract

Gastric cancer (GC) is among the most common and deadliest types of cancer, with a poor prognosis primarily due to late-stage detection and the presence of cancer stem cells (CSCs). This study investigates the mechanisms regulating extracellular adenosine levels in gastric cancer stem-like cells (GCSCs) derived from the MKN-74 cell line. Our results show that GCSCs release more ATP into the extracellular medium and exhibit higher levels of CD39 expression, which enables them to hydrolyze a greater amount of ATP. Furthermore, we also found that GCSCs possess a greater capacity to hydrolyze AMP, primarily due to the activity of the CD73 protein, with no significant changes in CD73 transcripts and protein levels between GCSCs and differentiated cells. Additionally, adenosine transport is primarily mediated by members of the equilibrative nucleoside transporter (ENT) family in GCSCs, where a significant increase in the expression level of the ENT2 protein is observed compared to non-GCSCs MKN-74 cells. These findings suggest that targeting the adenosine metabolism pathway in GCSCs could be a potential therapeutic strategy for gastric cancer.

## 1. Introduction

Gastric cancer (GC), also referred to as stomach cancer, is among the most common types of cancer. It currently ranks as the fifth most prevalent cancer and is the fourth leading cause of cancer-related deaths globally [1]. Despite improvements in diagnosis and treatment, the prognosis for patients with gastric cancer remains grim, primarily due to late-stage detection, which restricts treatment options and necessitates palliative care [2]. A study shows that 38.8% of patients with gastric cancer in Europe experience a recurrence within two years after surgery [3]. In recent years, a decline in the incidence and mortality of gastric cancer has been noted worldwide. However, the incidence rate has increased among adults under 50 years of age [4].

Studies indicate that one reason for this unfavorable prognosis is the presence of cancer stem cells (CSCs) [5]. These CSCs represent only a small fraction of the cells that make up the tumor. They display characteristics such as self-renewal, clonal tumorigenesis, differentiation, and long-term repopulation [5,6]. In the case of gastric cancer (GC), studies indicate that gastric cancer stem cells are responsible for phenomena such as progression, recurrence, metastasis, and drug resistance in this type of cancer [5]. Although it is true that there are advances in the study of these cell populations, the mechanisms by which these GCSCs promote recurrence, progression, metastasis, and drug resistance in GC are not entirely clear to date [7].

Various investigations in recent years indicate that adenosine, an endogenous nucleoside, plays a significant role in the tumor microenvironment [8,9,10]. The increase in extracellular adenosine levels has been associated with phenomena such as cell migration, invasion, and chemoresistance in cancer stem cells (CSCs) [11,12]. Extracellular adenosine levels in both healthy and tumor tissues are regulated primarily by two complementary mechanisms. First, the ectonucleotidases CD39 and CD73 metabolize extracellular ATP to generate adenosine [13]. As mentioned before, once it is extruded from the cells, ATP is dephosphorylated to AMP through the action of the CD39 protein. The AMP is subsequently converted into adenosine by the activity of the CD73 enzyme [14]. Second, transmembrane adenosine transport is mediated by two families of transporters, concentrative nucleoside transporters (CNTs) and equilibrative nucleoside transporters (ENTs), which modulate extracellular adenosine by facilitating its uptake into cells, where it can be further metabolized [15]. ENT1 and ENT2 are classified as equilibrative nucleoside transporters, which are part of the SLC29 family. Their function involves the bidirectional transport of nucleosides and nucleobases (such as adenosine and hypoxanthine) across cellular membranes, which is essential for nucleotide salvage pathways and the uptake of nucleoside-derived pharmaceuticals (including gemcitabine and 5-fluorouracil) [16].

However, there is no information about this in GCSCs. Therefore, understanding the cellular mechanisms that regulate extracellular adenosine levels in GCSCs could lead to the development of new, personalized therapies targeting these cells. Our objective is to investigate whether GCSCs display elevated levels of extracellular adenosine and the extracellular proteins that promote this process.

## 2. Results

### 2.1. Gastric Cancer Stem Cells (GCSCs) Have an Intrinsically Increased Capability to Generate Extracellular Adenosine

**Figure 1:**
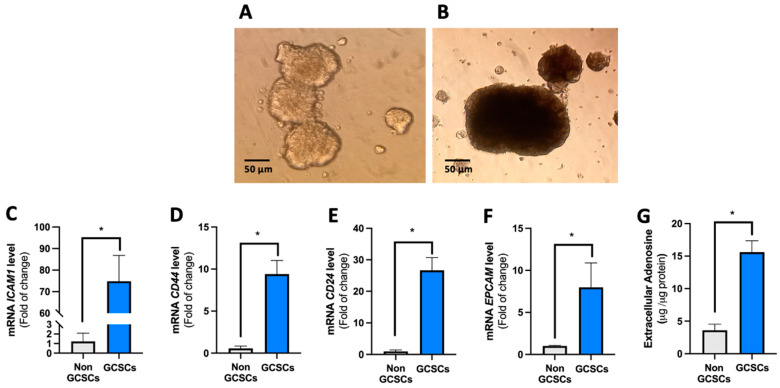
The gastric cancer stem cells (GCSCs) exhibit a high level of extracellular adenosine. MKN-74 cells were subjected to various culture conditions to cultivate GCSCs and non-GCSCs, as delineated in the Methods section. (**A**) Spheres at 3 days and (**B**) 7 days; light microscopy images of spheres (GCSCs) derived from the MKN-74 cell line. Quantitative reverse transcription polymerase chain reaction (qRT-PCR) analysis of (**C**) *ICAM1*, (**D**) *CD44*, (**E**) *CD24*, and (**F**) *EPCAM* in GCSCs and non-GCSCs. Values were normalized to *ACTB* mRNA levels. (**G**) The levels of extracellular adenosine were quantified in the culture medium, as described in Section 4.5. The plots represent the means ± SD. * *p* < 0.05. *n* = 5.

When maintained in serum-free medium, the MKN-74 cell line was capable of forming non-adherent cell clusters or spheres, a cell subpopulation enriched in gastric cancer stem cells (GCSCs) [17]. Elevated levels of adenosine have been observed in the tumor microenvironment, where cancer stem cells reside [15,18]. However, no information is available about the level of adenosine in GCSCs. To respond to this, GCSCs were generated from the MKN-74 cell line, as detailed in Section 4.3 (Figure 1A,B) [17]. To verify that these structures display STEM characteristics, qRT-PCR assays were performed to assess the transcript levels of the *ICAM1*, *CD44*, *CD24*, and *EPCAM* genes. We determined that GCSCs present high levels of transcripts with respect to differentiated MNKN-74 cells for all the genes described above (Figure 1C–F). Later, we determined the level of extracellular adenosine as described in Section 4.5. For this, we used GCSCs derived from the MKN-74 cell line as our model and compared them with MKN-74 cells under differentiating conditions. We found that MKN-74 GCSCs have 5 times more extracellular adenosine compared to their differentiated cells (Figure 1G). In conclusion, MKN-74 GCSCs demonstrate elevated levels of extracellular adenosine relative to differentiated cells.

### 2.2. Gastric Cancer Stem Cells (GCSCs) Have a High Capacity to Degrade ATP

**Figure 2:**
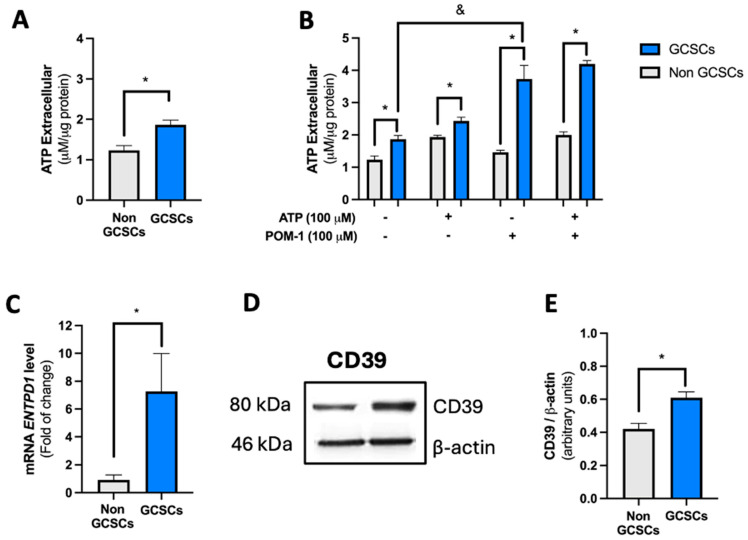
The gastric cancer stem cells (GCSCs) exhibit a high capacity to hydrolyze ATP. MKN-74 cells were subjected to different culture conditions to generate GCSCs and non-GCSCs, as detailed in the Materials and Methods (Section 4). (**A**) Extracellular ATP levels were quantified in the culture medium as described in Section 4.4. (**B**) Extracellular ATP levels were quantified in the culture medium exposed to 100 µM ATP and/or 100 µM POM-1 (to inhibit CD39). (**C**) qRT-PCR analysis of *ENTPD1* in GCSCs and non-GCSCs. Values were normalized to *ACTB* mRNA. (**D**) Representative Western blot of CD39 and β-actin in GCSCs and non-GCSCs. (**E**) The graph represents the quantification of CD39 signals in Western blot normalized against β-actin signals. The plots represent the means ± SD. * *p* < 0.05. *n* = 4.

**Figure 3:**
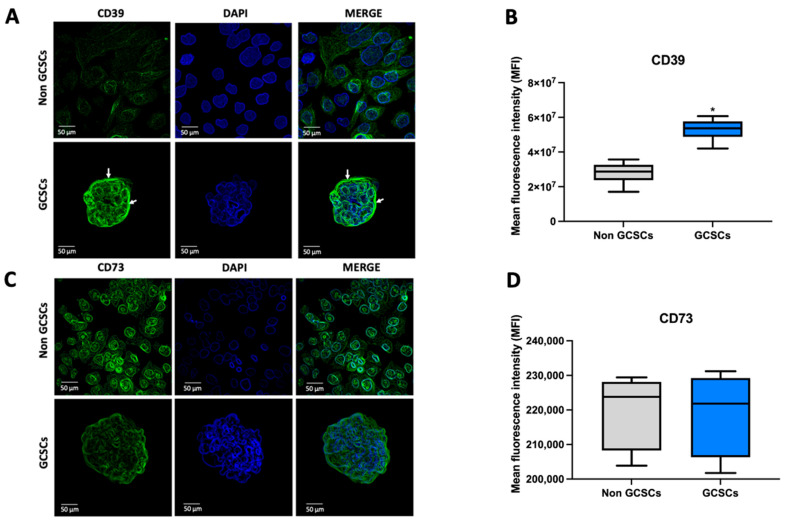
Immunofluorescence staining for CD39 and CD73. (**A**) Representative images of spheroids derived from MKN-74 cells, compared to adherent MKN-74 cells. The expression of CD39 and (**C**) CD73 was visualized using a green fluorescent dye, while cell nuclei were counterstained with DAPI. Scale bar: 50 μm. (**B**,**D**) show the quantification of mean fluorescence intensity (MFI) for CD39 and CD73, respectively, in both cell types. Data represent the analysis of six representative images from distinct fields per condition, obtained from three independent experiments. Graphs show means ± SD. * *p* < 0.05; *n* = 6.

As mentioned in the introduction, cells regulate extracellular adenosine levels through various mechanisms. One of these mechanisms is by modulating their ability to hydrolyze ATP. In this context, our first approach was to evaluate the level of extracellular ATP produced by GCSCs derived from the MKN-74 cell line and compare it with that of MKN-74 cells under differentiating conditions. The determination of extracellular ATP levels was conducted as described in Section 4.4. We found that MKN-74 GCSCs release double the amount of extracellular ATP when compared to their differentiated counterparts (Figure 2A). Later, we conducted ATP overload assays to assess the activity of ATP-metabolizing enzymes exhibited by MKN-74 GCSCs and differentiated MKN-74 cells. We observed that when ATP was added, both differentiated cells and GCSCs increased the extracellular level of this nucleotide. However, in both cases, this increase only reflected a fraction of the added nucleotide, indicating that in both conditions, these cells possess a high capacity to hydrolyze ATP (Figure 2B). Later, an ATP overload assay was performed to assess the function of the CD39 protein as described in the Materials and Methods (Section 4). The graph shows the difference between the total ATP initially loaded and the ATP remaining after each treatment. Incubation with the exogenous substrate caused a significant increase in extracellular ATP in both non-GCSCs and GCSCs. Subsequently, when the CD39 inhibitor (POM-1) was used, it was observed that in GCSCs, ATPase activity was strongly dependent on this enzyme’s activity, evidenced by the rise in extracellular ATP levels compared to non-GCSCs, where no significant increase was observed after POM-1 treatment. This indicates that CD39 plays an important role in GCSCs in their ability to degrade ATP compared to non-GCSCs (Figure 2B). Subsequently, the transcript level of the *ENTPD1* gene, which encodes the CD39 protein, was evaluated in GCSCs and non-GCSCs of the MKN-74 cell line using qRT-PCR as described in Section 4.7. We observed a marked increase in the transcript level of the *ENTPD1* gene in GCSCs compared to non-GCSCs of the MKN-74 cell line (Figure 2C). Western blot analyses were developed as described in Section 4.6. The experiments demonstrated that GCSCs had a greater expression of CD39 protein than non-GCSCs in the MKN-74 cell line (Figure 2D,E). Finally, we performed immunofluorescence assays as described in Section 4.10. To corroborate the results obtained from Western blot analysis, immunofluorescence analyses were conducted to evaluate the expression of CD39 in GCSCs. CD39 expression levels were significantly higher in spheroids compared to adherent MKN-74 cells. CD39 was predominantly localized at the cell membrane in spheroids, as shown by white arrows, while adherent cells displayed lower fluorescence intensity (Figure 3A,B). Evidence indicates that GCSCs release a greater quantity of ATP into the extracellular media compared to non-GCSCs from the MKN-74 line. Moreover, they demonstrate enhanced expression of the CD39 protein, allowing GCSCs to hydrolyze ATP more efficiently than non-GCSCs of the MKN-74 cell line.

### 2.3. Gastric Cancer Stem Cells Present Greater AMPase Capacity than Non-GCSCs of the MKN-74 Cell Line

**Figure 4:**
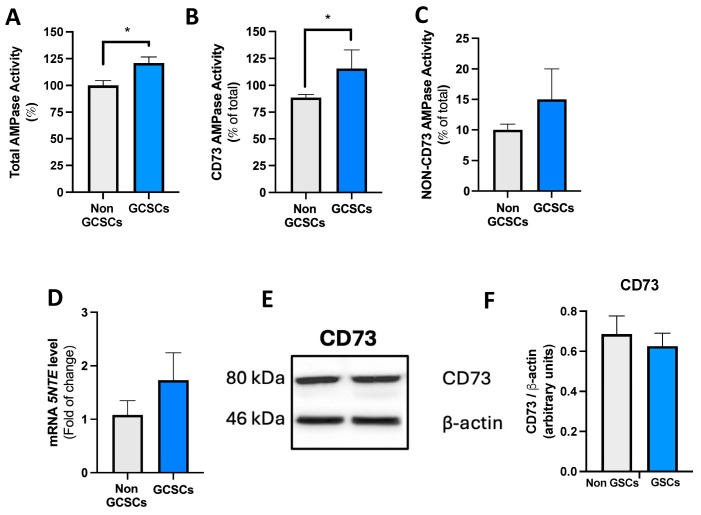
Expression and activity of the AMP-metabolizing enzyme CD73 in gastric cancer stem cells. (**A**) Total, (**B**) CD73, and (**C**) non-CD73 AMPase activity were evaluated in GCSCs and non-GCSCs derived from the MKN-74 cell line, as described in Section 4.9. (**D**) Relative transcript levels of the *5NTE* gene in GCSCs and non-GCSCs. Values were normalized to *ACTB* mRNA expression. (**E**) Representative Western blot of CD73 and β-actin in GCSCs and non-GCSCs. (**F**) The graph represents the quantification of the CD73 signal in Western blot normalized against the β-actin signal. The graphs represent the means ± SD. * *p* < 0.05. *n* = 6.

In the study of extracellular metabolism of adenosine in our working model, the next step was to evaluate the capacity of GCSCs and non-GCSCs from the MKN-74 cell line to hydrolyze AMP. Initially, we evaluated the AMPsa capacity in the previously described cells, as outlined in Section 4.9. We found that GCSCs exhibit a higher total AMPase capacity compared to non-GCSCs generated from the MKN-74 cell line (Figure 4A). One of the primary cellular components involved in extracellular AMP hydrolysis is the CD73 protein. In this context, we began by evaluating the AMPase activity associated with the CD73 protein in GCSCs and non-GCSCs of the MKN-74 cell line. Both in GCSCs and non-GCSCs, we determined that the AMPase capacity these cells exhibit is largely attributed to the CD73 protein (Figure 4B). On the other hand, we determined that in GCSCs and non-GCSCs, lower AMPase capacity in these structures is not dependent on the CD73 protein (Figure 4C). The transcript level of the *NT5E* gene, which encodes the CD73 protein, was subsequently evaluated in GCSCs and non-GCSCs of the MKN-74 cell line using qRT-PCR as described in Section 4.7. We determined that there are no significant changes in the transcript level of the *NT5E* gene between GCSCs and non-GCSCs of the MKN-74 cell line (Figure 4D). Subsequently, the level of CD73 protein was assessed in the cellular structures mentioned above, as detailed in Section 4.10. In these assays, we determined no significant differences at the protein level between GCSCs and non-GCSCs (Figure 4E,F). To corroborate the results obtained by Western blot, immunofluorescence analyses were performed to evaluate the expression of CD73 in GCSCs. CD73 expression appeared more diffuse in spheroids, lacking a predominant membrane localization (Figure 3C). Quantification of fluorescence intensity revealed no significant differences in CD73 expression between spheroids and adherent cells (Figure 3D), suggesting that CD73 is not differentially expressed under these culture conditions. Based on the evidence presented, we conclude that GCSCs exhibit a higher total AMPase capacity compared to differentiated MKN-74 cells. The AMP capacity in both non-GCSCs and GCSCs is primarily driven by the action of the CD73 protein. The increase in AMPase capacity in GCSCs is likely due to enhanced activity of this protein in cancer stem cells.

### 2.4. Gastric Cancer Stem Cells Have a Higher Level of ENT2 than Non-GCSCs of the MKN-74 Cell Line

**Figure 5:**
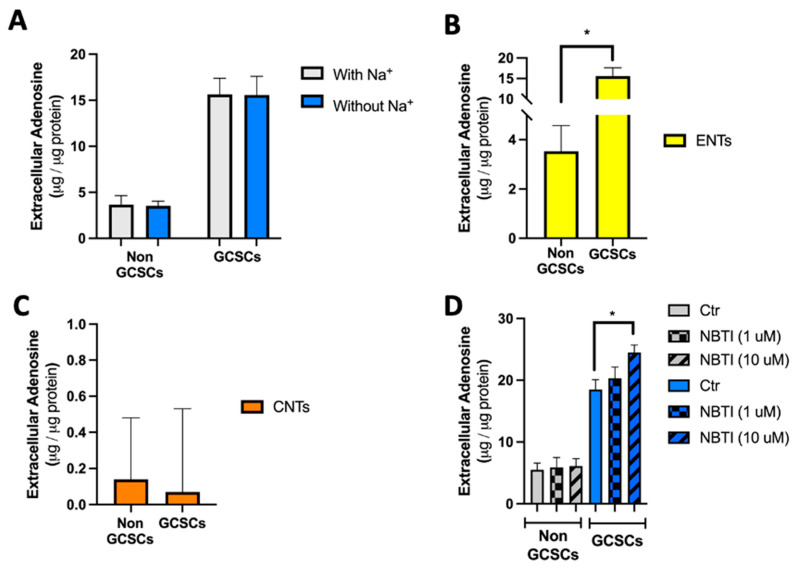
ENTs regulate adenosine transport in both GCSCs and non-GCSCs. Adenosine accumulation assays were conducted as outlined for adenosine in Section 4.8. (**A**) Extracellular adenosine levels were determined in Tyrode’s medium both with and without NaCl. (**B**) Extracellular adenosine levels were measured in Tyrode’s medium without NaCl. (**C**) Extracellular adenosine levels were measured in Tyrode’s medium with NaCl. (**D**) Extracellular adenosine levels were quantified in culture medium exposed to 1 µM NBTI (to inhibit ENT1) or 10 µM NBTI (to inhibit ENT1 and ENT2). The plots represent the means ± SD. * *p* < 0.05. *n* = 5.

According to the results previously described in this article, GCSCs generated from the MKN-74 cell line produce a significantly greater amount of extracellular adenosine than differentiated MKN-74 cells or non-GCSCs. To clarify which cellular mechanisms contribute to this, our next step was to perform assays to assess the role of concentrative nucleoside transporters (CNTs) and equilibrative nucleoside transporters (ENTs) in regulating extracellular adenosine levels. To achieve this, adenosine accumulation assays were developed as detailed in Section 4.8. We employed GCSCs derived from the MKN-74 cell line as previously described. We determined that MKN-74, derived from GCSCs, accumulates five times more adenosine than MKN-74 cells (Figure 1D). Following this, we assessed the roles of CNTs and ENTs within the described context in GCSCs and non-GCSCs of the aforementioned cell line. We determined that in both cases, the accumulation of extracellular adenosine was unaffected by the presence or absence of sodium in the medium (Figure 5A). Data analysis revealed that the accumulation of extracellular adenosine in GCSCs and non-GCSCs relies on the function of ENTs (Figure 5B). We also determined that there are no significant differences in the action of concentrative nucleoside transporters (CNTs) between GCSCs and non-GCSCs (Figure 5C). Finally, adenosine accumulation assays were performed in the presence of NBTI at different concentrations to inhibit ENT1 and ENT2 transporters (Figure 5D). After inhibition of ENT1-mediated uptake using 1 μM NBTI, extracellular adenosine levels in GCSCs were not significantly higher compared to the control. When inhibiting transport by ENT1 and ENT2 using 10 μM NBTI, we observed an increase in extracellular adenosine levels compared to the control, with no significant changes in the content of extracellular adenosine when ENT1-mediated transport alone was inhibited.

**Figure 6:**
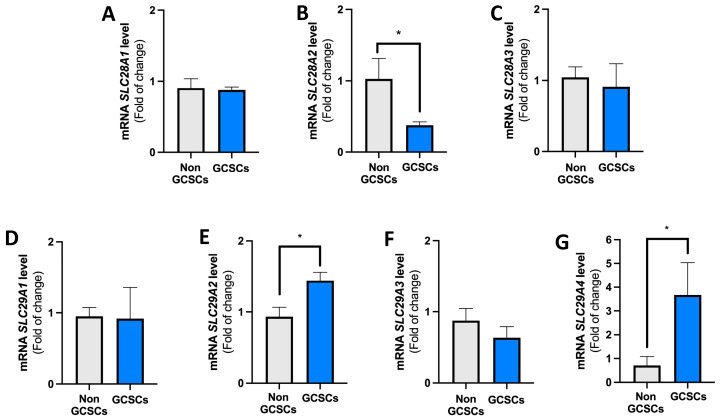
Transcript levels of genes encoding CNTs and ENTs in GCSCs and non-GCSCs. MKN-74 cells were subjected to different culture conditions to generate GCSCs and non-GCSCs as described in the Methods section. qRT-PCR of (**A**) *SLC28A1*, (**B**) *SLC28A2*, (**C**) *SLC28A3*, (**D**) *SLC29A1*, (**E**) *SLC29A2*, (**F**) *SLC29A3* and (**G**) *SLC29A4* in GCSCs and non-GCSCs. Values were normalized to ACTB mRNA. The plots represent the means ± SD. * *p* < 0.05. *n* = 5.

Later, the transcript levels of the genes encoding CNT1 (*SLC28A1*), CNT2 (*SLC28A2*), CNT3 (*SLC28A3*) (Figure 6A–C), ENT1 (*SLC29A1*), ENT2 (*SLC29A2*), ENT3 (*SLC29A3*), and ENT4 (*SLC29A4*) (Figure 6D–G) in GCSCs and non-GCSCs were assessed using qRT-PCR. Regarding the transcripts of genes that encode CNTs, a significant decrease was observed in the number of transcripts for the gene *SLC28A2* (Figure 6B). No significant differences in transcript levels were observed between GCSCs and non-GCSCs for the *SLC28A1* (Figure 6A) and *SLC28A3* (Figure 6C) genes. In analyzing the transcripts of genes encoding members of the ENT family, we observed that there were no significant changes in the levels of *SLC29A1* (Figure 6D) and *SLC29A3* (Figure 6F) gene transcripts between GCSCs and non-GCSCs. Additionally, we noted an increase in the levels of transcripts for the *SLC29A2* and *SLC29A4* genes in GCSCs compared to non-GCSCs (Figure 6E,G).

**Figure 7:**
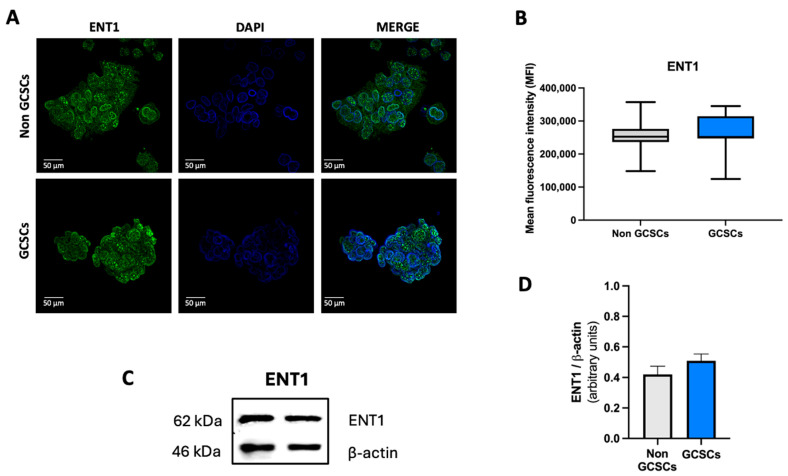
ENT1 expression in gastric cancer stem cells (GCSCs). (**A**) Representative images of spheroids derived from MKN-74 cells, compared to adherent MKN-74 cells. ENT1 expression was visualized using a green fluorescent dye, while cell nuclei were counterstained with DAPI. Scale bar: 50 μm. (**B**) Quantification of mean fluorescence intensity (MFI) of ENT1 signal in both cell types. Data represent the analysis of six representative images from distinct fields per condition, obtained from three independent experiments. Graphs show means ± SD; *n* = 6. (**C**) Representative Western blot of ENT1 and β-actin in GCSCs and non-GCSCs. (**D**) Quantification of ENT1 signal from Western blot normalized to β-actin. Bars represent means ± SD. * *p* < 0.05; *n* = 5.

**Figure 8:**
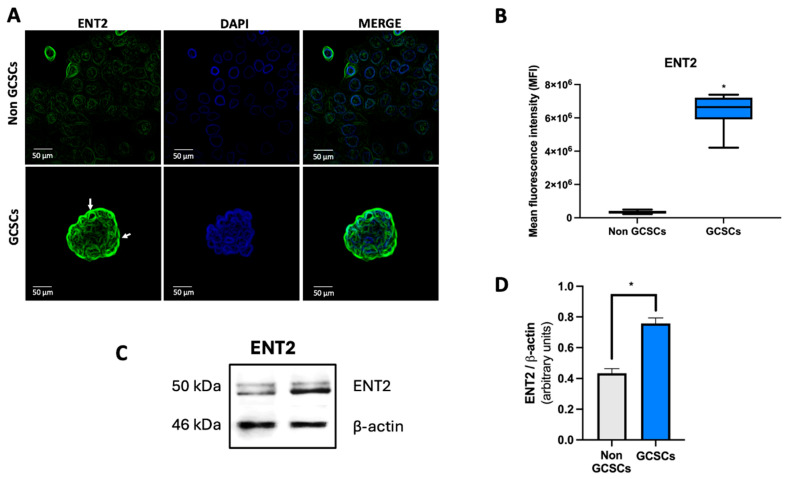
GCSCs exhibit higher levels of ENT2 compared to non-GCSCs. (**A**) Representative images of spheroids derived from MKN-74 cells, compared to adherent MKN-74 cells. ENT2 expression was visualized using a green fluorescent dye, while cell nuclei were counterstained with DAPI. Scale bar: 50 μm. (**B**) Quantification of mean fluorescence intensity (MFI) of ENT2 signal in both cell types. Data represent the analysis of six representative images from distinct fields per condition, obtained from three independent experiments. Graphs show means ± SD; *n* = 6. (**C**) Representative Western blot of ENT2 and β-actin in GCSCs and non-GCSCs. (**D**) Quantification of ENT2 signals from Western blot normalized to β-actin. Bars represent means ± SD. * *p* < 0.05; *n* = 3.

Western blot analysis was conducted to assess the levels of ENT1 and ENT2 proteins in non-GCSCs. Through this technique, we determined that there are no significant differences in the level of ENT1 protein among the structures described above (Figure 7C,D). In contrast, for ENT2, we found that GCSCs exhibit a significantly higher level of this protein compared to differentiated MKN-74 cells (Figure 8C,D). Finally, we performed immunocytochemistry assays as described in Section 4.10. To corroborate the results obtained by Western blot, immunofluorescence analyses were conducted to evaluate the expression of ENT1 and ENT2 in GCSCs (Figure 7A and Figure 8A). Immunofluorescence analysis revealed that ENT1 was expressed in both spheroids and adherent MKN-74 cells; however, its distribution appeared more diffuse in spheroids, lacking clear membrane localization. Representative images show ENT1 expression (green) with nuclear counterstaining using DAPI (blue) (Figure 7A). Quantification of mean fluorescence intensity demonstrated no significant differences between spheroids and adherent cells (Figure 7B). On the other hand, ENT2 expression levels were markedly increased in spheroids compared to adherent MKN-74 cells. As indicated by white arrows, ENT2 was predominantly localized at the cell membrane in spheroids, while adherent cells exhibited lower fluorescence intensity (Figure 8A). Quantifying mean fluorescence intensity demonstrated significant differences between spheroids and adherent cells (Figure 8B) around the ENT2 protein. We found that the transport of adenosine is facilitated by both non-GCSCs and GCSCs through transporters that are members of the ENTs family, according to the evidence provided. Furthermore, we determined that GCSCs exhibit a higher expression of the ENT2 protein in comparison to non-GCSCs.

## 3. Discussion

Gastric cancer (GC) is currently the fourth leading cause of cancer-related deaths worldwide [19]. Evidence suggests that the limited effectiveness of current treatments for this condition results from the presence of GCSCs [20]. The tumor microenvironment (TME), where CSCs reside, plays a crucial role in cancer progression, and adenosine, a metabolite produced by cancer cells, significantly influences this environment, especially concerning CSCs. Adenosine, produced through ATP breakdown, contributes to tumor progression and suppression of the immune response by activating adenosine receptors expressed on various cell types within the tumor microenvironment (TME) [21]. To date, there are no studies on the extracellular levels of adenosine in GCSCs. Evidence shows that this nucleoside is crucial in shaping the tumor microenvironment (TME) and promoting metastasis. It acts as an immunosuppressant, inhibiting the antitumor immune response and creating an environment conducive to tumor growth and spread [9,10,21]. In this way, understanding the cellular mechanisms by which GCSCs modulate their extracellular adenosine levels could lead to new therapeutic strategies for managing this disease.

Studies indicate a rise in extracellular adenosine levels in various CSCs of different types of cancer [22,23]. In this context, we chose to investigate what occurs in the case of GCSCs generated from the MKN-74 cell line. In this study, we demonstrated that GCSCs exhibit higher extracellular adenosine levels than non-GCSCs from the MKN-74 cell line. This finding aligns with findings from other studies on CSC [18,22,23]. CSCs derived from glioblastoma (GSCs) exhibit elevated levels of extracellular adenosine compared to differentiated cells [15,22]. A similar phenomenon has been observed in CSCs derived from non-small cell lung cancer (NSCLC) cell lines H3122 and A549 [24]. The increase in the level of extracellular adenosine in CSCs has been associated with phenomena such as chemoresistance, cell invasion, and migration [12,24,25,26]. Consequently, our subsequent step involved examining some cellular mechanisms that regulate the extracellular concentration of adenosine in the produced GCSCs.

One of the pathways through which adenosine can be generated is via extracellular ATP (eATP) metabolism, a process mediated by the CD39/CD73 enzymes both under normal conditions and in cancer [27]. CD39 is an integral membrane protein that metabolizes eATP to AMP. Increased expression of this protein has been described in various types of neoplasia, such as hepatocellular carcinoma, melanoma, leukemia, and lung, gastric, colorectal, ovarian, breast, head, neck, and rectal cancer [28]. In our study, we describe that GCSCs exhibit elevated levels of the CD39 protein compared to differentiated cells. To date, there are few studies evaluating the level of CD39 in CSCs; however, one study indicates that CD39 is expressed at high levels in glioma cancer stem cells compared to non-stem glioma cells [29]. In addition, we determined that the extracellular ATP level in MKN-74 cells is in the µM range, which correlates with findings by Coddou [30]. This article indicates that the AGS, MKN-45, and MKN-74 cell lines exhibit high levels of extracellular ATP compared to the non-tumoral gastric cell line GES-1 [30]. Our study indicates that GCSCs derived from the MKN-74 cell line exhibit higher eATP levels than differentiated MKN-74 cells. In several cellular models, eATP has been shown to induce processes such as angiogenesis, immunosuppression, proliferation, migration, and invasion [31,32,33,34]. Interestingly, eATP has been reported to induce the formation of CSCs in human non-small cell lung cancer (NSCLC) cells [35]. In this context, no information is available on the effect of eATP on GCSCs. Interestingly, studies have shown that high expression of CD39 correlates with unfavorable prognoses in gastric cancer in human samples [36,37]. However, there are also reports suggesting that elevated levels of CD39 expression are linked to a favorable prognosis in other cancer types, such as lung adenocarcinoma [38] and pancreatic cancer [39]. Thus, the potential impact of CD39 on cancer progression will be determined by the varying expression of this protein in the various cell types that constitute the tumor.

As mentioned above, the literature indicates that CD73 is a crucial protein in the extracellular adenosine generation pathway. Elevated CD73 expression has been linked to poor prognosis in breast, ovarian, lung, gastric, colorectal, prostate, and melanoma cancers [24,40]. However, in endometrial carcinoma or clear cell renal carcinoma, an increase in CD73 expression seems to be a protective factor [41,42]. CD73 has been described to be involved in sphere formation and tumor initiation in ovarian carcinoma cells [43]. Our results indicate an increase in AMPase activity in GCSCs of the MKN-74 cell line compared to non-GCSCs, with no significant changes in CD73 transcript and protein levels. Spheroids (cancer stem cells) derived from the 786-O cell line (renal carcinoma) have been described [44] CeCa (cervical cancer) [45], H3122 (non-small-cell lung cancer) [24], and A549 (lung cancer) [24] exhibit an elevated level of the CD73 protein compared to the cell line from which they are derived. However, there is also evidence in the literature showing instances where spheroids derived from the cell line H2228 (Human Lung Cancer Cells) [24] do not show significant changes in the level of the CD73 protein compared to the cell line from which they originated. This last point correlates with the results obtained by our team. A study on various gastric cancer cell lines demonstrates that hypoxia induces CD73 expression [46]. Various studies indicate that CSCs inhabit hypoxic niches within tumors [47]. Research is necessary to determine if spheroids exposed to hypoxia show higher levels of CD73 compared to the originating cell line.

A notable finding in our study is that CD73 enzymatic activity is elevated in gastric cancer stem cells (GCSCs), even though no significant differences were observed in either NT5E transcript levels or CD73 protein levels, as determined by Western blot or immunofluorescence. This apparent disconnect between expression and function indicates that CD73 regulation in GCSCs may occur post-transcriptionally or based on cellular context, rather than through increased transcription. This could be because the subcellular localization of CD73 enhances its catalytic activity within GCSCs. Although the immunofluorescence appears diffuse, a functionally important pool of CD73 might be relocated to specific membrane areas, such as basolateral protrusions, where access to AMP substrates is more accessible. Another possibility involves post-translational modifications, like different glycosylation patterns. CD73 is a GPI-anchored ectonucleotidase whose activity can be influenced by glycan structures, particularly sialylation, or interactions with other membrane proteins [48,49,50]. These modifications could enhance CD73’s stability or catalytic efficiency without altering total protein levels. Additionally, the acidic microenvironment of solid tumors may contribute to the observed increase in CD73 activity in GCSCs. Previous reports indicate that low extracellular pH can promote AMP-to-adenosine conversion by stabilizing the active conformation of CD73 and prolonging adenosine half-life [46,51]. This mechanism could help explain why GCSCs show elevated AMPase activity despite unchanged CD73 expression. These results indicate that CD73 activity in GCSCs is intricately regulated by context-dependent mechanisms, such as membrane localization, post-translational modifications, and extracellular pH, which may not be accurately captured by expression levels alone. Additional studies are needed to elucidate the contribution of these regulatory layers to CD73 functionality.

Nucleoside transporters are essential for modulating extracellular adenosine levels and, consequently, its availability to interact with its receptors. Evidence indicates that both concentrative and equilibrative nucleoside transporters (ENTs and CNTs) play a crucial role in regulating tumor adenosine levels [52]. We concentrated on ENTs because our data indicate they are the main contributors, over CNTs, to extracellular adenosine accumulation in the studied models. Although a statistically significant downregulation was only observed for SLC28A2 (Figure 6B), our analysis shows a consistent trend toward reduced transcript levels for all three CNT family members (SLC28A1–3) in GCSCs compared to differentiated MKN-74 cells (Figure 6A–C). Although not previously discussed in this manuscript, it is well established that CNT2 and CNT3 exhibit higher affinity for adenosine compared to CNT1 and the ENT family of transporters. However, several reports have shown that CNT3 expression is limited across various cell types, suggesting that adenosine uptake via concentrative transport is predominantly mediated by CNT2 in most cellular contexts [52]. Studies indicate that CNT2 and CNT3 transporters exhibit a greater affinity for adenosine than ENT proteins [53]. However, despite a decrease in transcript levels being observed, there are no significant changes in the accumulation of adenosine in the extracellular space mediated by these transporters. The CNT2 protein has been described as being highly expressed in the gastrointestinal tract. A study indicates elevated levels of SLC28A2 mRNA in the stomach [54]. However, low levels of this protein have been observed in tumor tissue samples from the stomach and other organs, including the kidneys, rectum, and small intestine [55]. Regarding studies with human biopsies, the evidence around CNT2 is controversial: while one study indicates that high expression of this protein correlates with a poor response to neoadjuvant concurrent chemoradiotherapy and worse patient outcomes for survival in rectal cancer [56], another study indicates that CNT2 expression is significantly repressed in colorectal cancer [57]. This discrepancy may result from the chemotherapy and/or radiotherapy treatments received by the patients who donated the biopsies. Therefore, it would be valuable in future studies to assess the expression and activity of the CNT2 protein in human gastric cancer biopsies.

In relation to ENTs, this family of transporters is composed of four members that move nucleosides in favor of a concentration gradient. Evidence the ENT1 and ENT2 proteins demonstrate distinct affinities in the transport of purine and pyrimidine nucleosides. Both proteins are predominantly found in the plasma membrane; however, the nuclear localization of ENT2 has been reported, where this protein would be related to the translocation of substrates to the nucleus during the process of DNA replication [55,58]. Regarding the other members of this family of transporters, ENT3 is reported to be located in the membranes of lysosomes and mitochondria [53]. Finally, ENT4 is described as being located in intracellular organelles and, to a lesser extent, in the plasma membrane [59]. Due to their cellular localization, the ENT1 and ENT2 proteins are linked to regulating the extracellular level of adenosine in various cellular contexts [52,53,58].

Our results indicate that GCSCs derived from the MKN-74 cell line do not exhibit significant differences in the expression of the ENT1 protein with respect to non-GCSCs. The expression of this protein has been reported in the MKN-74 cell line, which correlates with our findings. However, there is no information regarding the expression of other members of the family of equilibrative and concentrative nucleoside transporters in this cell line [60]. This study indicates that total ENT1 protein levels are not altered in 5-Fluorouracil (5-FU) resistant MKN-74 cells. Therefore, resistance to this chemotherapeutic agent is not mediated by changes in this protein level [60]. A study reveals that the downregulation of ENT1 is one of the mechanisms through which gemcitabine resistance develops in pancreatic cancer [61]. Despite the results of our study, we cannot rule out the involvement of ENT1 in certain phenomena that contribute to the chemoresistance observed in stomach cancer.

At the transcript level, the stomach has been identified as having high expression of the ENT1 and ENT2 proteins [54]. Studies show that ENT1 is widely expressed in the stomach, while ENT2 is expressed in both the stomach and duodenum [59]. Likewise, several studies have reported elevated levels of ENT2 in various cancers originating from digestive tract organs. A glioblastoma stem cells (GSCs) study demonstrates differing expressions of ENT1 and ENT2 in different populations of these cancer stem cells [15]. This study shows that mesenchymal GSCs exhibit lower ENT1 expression and activity, which correlates with higher extracellular adenosine levels than proneural GSCs [15]. Our study also finds that GCSCs have more of the ENT2 protein than the differentiated MKN-74 cells. Evidence suggests that this protein can transport purines and pyrimidines. Its ability to translocate hypoxanthine is reported to be significantly greater than that of ENT1 [58,62]. A study of colorectal cancer (CRC) cell lines demonstrates a rise in ENT2 expression compared to normal cells, which correlates with increased hypoxanthine transport for DNA synthesis [63]. Notably, 5-FU, one of the main drugs used to treat stomach cancer, has been identified as a substrate for ENT1 and ENT2 proteins [64,65]. Evidence indicates that the primary cause of 5-FU resistance has been attributed to the presence of cancer stem-like cells (CSCs) within the cancer cell niche [66]. Based on this, ENT2 protein may be a promising target for developing new therapies aimed at various cell types that exhibit high expression of this protein. Regarding ENT4, our results show a significant increase in the transcript level of the SLC29A4 gene in GCSCs compared to non-GCSCs. SLC29A4, also known as PMAT (Plasma Membrane Monoamine Transporter), is a protein that transports various organic cations and monoamines across cell membranes. It has also been reported to transport nucleosides and drugs [67,68]. Studies indicate that this protein is highly expressed in neuroblastoma cells and can influence the transport of meta-iodobenzylguanidine (mIBG), which is used in targeted cancer therapy [68]. To date, there are no antecedents regarding the expression or activity of this protein in gastric cancer biopsies.

This study shows that spheroids derived from the MKN-74 cell line have notably higher extracellular adenosine levels compared to those generated by differentiated MKN-74 cells. Our findings show that GCSCs obtained from the MKN-74 cell line possess an increased ability to hydrolyze ATP and AMP compared to differentiated cells. This is partly due to an increase in CD39 expression, a protein that converts ATP to AMP in the extracellular space, along with a rise in AMPase capacity associated with the CD73 protein in GCSCs. Finally, we demonstrate that these GCSCs exhibit significantly higher levels of the ENT2 protein compared to differentiated cells. The evidence presented in this article indicates that GCSCs have mechanisms that enable them to produce and accumulate more adenosine in the extracellular space than differentiated cells of the MKN-74 lineage. Increased extracellular adenosine levels have been associated with phenomena such as chemoresistance, cell invasion, migration, and recurrence in various types of cancer. Therefore, examining the biological implications of the elevated levels of extracellular adenosine observed in GCSCs could clarify the mechanisms associated with progression, metastasis, and treatment resistance seen in gastric cancer. Although our findings are valuable, it is important to remember that a cell line comes from a specific type of cancer, so we cannot apply our results broadly to stomach cancer. Future research should include other stomach cancer cell models and human stomach cancer samples.

## 4. Materials and Methods

### 4.1. Pharmacological Agents

For in vitro studies, the following compounds were used: ATP (Cat# 987-65-5, Cayman Chemical Company, Ann Arbor, MI, USA), POM-1 (Cat# 314075-43-9, Cayman Chemical Company, Ann Arbor, MI, USA), Erythro-9-(2-hydroxy-3-nonyl) adenine (EHNA) (Cat#13352, Cayman Chemical Company, Ann Arbor, MI, USA), and PSB-12379 (Cat#28446, Cayman Chemical Company, Ann Arbor, MI, USA).

### 4.2. Cell Culture

The MKN-74 gastric cancer cell line was provided by the Armando Rojas Laboratory. It was grown in differentiation RPMI 1640 (with L-glutamine and HEPES 25mM) (Cat#SH30255.FS, Cytiva, Marlborough, MA, USA) medium supplemented with 10% heat-inactivated fetal bovine serum and penicillin-streptomycin (Life Technologies, Carlsbad, CA, USA) in standard culture conditions (37 °C and 5% CO_2_).

### 4.3. Gastric Stem-like Cell Culture

For the generation of GCSCs from the MKN-74 cell line, the cells were grown in RPMI 1640 medium (with L-glutamine and HEPES 25 mM) (Cat#SH30255.FS, Cytiva, Marlborough, MA, USA) supplemented with EGF (20 ng/mL; Peprotech^©^, Rocky Hill, NJ, USA), bFGF (10 ng/mL; Peprotech^©^), 1X B27 (Gibco™) and penicillin/streptomycin (100 U/mL, Gibco) at 37 °C. After 5 days of culture, GCSCs were plated to carry out different tests and treatments. For expansion, GCSCs were washed with PBS 1X and then treated for 10 min at 37 °C with StemPro^®^ Accutase^®^ (ThermoFisher, Waltham, MA, USA). Subsequently, the cells were maintained in the medium described above for the generation of new GCSCs.

### 4.4. ATP Quantification

The MKN-74 cell line and GCSCs were maintained under standard culture conditions (37 °C, 5% CO_2_) for 5 days. Then, GCSCs and non-GCSCs were washed with PBS 1X two times and incubated in 500 µL of Tyrode’s buffer for 1 h at 37 °C. Later, 200 µL of this incubation medium was mixed with 100 µL of citrate buffer (pH 4). Subsequently, the level of ATP was determined using the ATP Detection Assay Kit-Luminescence (Cat#700410, Cayman Chemical Company, Ann Arbor, MI, USA) following the manufacturer’s recommendations. ATP concentrations (µM) were normalized to the total protein levels (µg).

For the ATP overload assays, the MKN-74 cell line and GCSCs were cultured under standard conditions (37 °C, 5% CO_2_) for 5 days. Then, they were exposed to 100 µM ATP (Cat# 987-65-5, Cayman Chemical Company, Ann Arbor, MI, USA) for 30 min in Tyrode buffer pH 6.0, with and without 100 µM POM-1 (Cat#314075-43-9, Cayman Chemical Company, Ann Arbor, MI, USA). Subsequently, ATP levels were measured as described in the previous paragraph.

### 4.5. Adenosine Quantification

The MKN-74 cell line and GCSCs were maintained under standard culture conditions (37 °C, 5% CO_2_) for 5 days. Then, GCSCs and non-GCSCs were washed with PBS 1X two times and incubated in 500 µL of Tyrode’s buffer for 1 h at 37 °C. Later, 200 µL of this incubation medium was mixed with 100 µL of citrate buffer (pH 4). Subsequently, the level of adenosine was determined using the Adenosine Quantification Assay Kit (MAK433-1KT, Sigma-Aldrich, Saint Louis, MO, USA) following the manufacturer’s recommendations. Adenosine concentrations (µM) were normalized to the total protein levels (µg).

### 4.6. Western Blots

Total protein extracts (40 µg) obtained from the MKN-74 cell line and GCSCs were fractionated by SDS-PAGE, transferred to 0.22 µm PVDF membranes (General Electric, GE^®^, Boston, MA, USA) and blocked with 1X PBS/0.05% Tween/1% BSA for 1 h. Then, membranes were incubated overnight with primary antibodies (Table S1) at 4 °C followed by a secondary antibody-HRP conjugate for 1 h. Western blot membranes were imaged using the LI-COR C-DiGit^®^ Blot Scanner (LI-COR Biosciences, Lincoln, NE, USA), which enables chemiluminescent detection and digital capture of protein bands. Western blots were revealed using the Clarity™ Western ECL Substrate (Cat# 170-5060, Bio-Rad Laboratories, Inc., Hercules, CA, USA) and images were quantified by densitometry analysis (ImageJ, software version 1.54p, NIH). Table S2 shows the densitometric analyses of these tests.

### 4.7. RNA Extraction and qRT-PCR

The MKN-74 cell line and GCSCs were maintained under standard culture conditions (37 °C, 5% CO_2_) for 5 days. Then, total RNA was extracted using TRIzol Reagent (Thermo Fisher Scientific, Waltham, MA, USA) and reverse transcription was performed with 0.5 µg of RNA using the High Capacity cDNA Reverse Transcription Kit (Cat#4368814, Applied Biosystems, Lithuania, Carlsbad, CA, USA), following the manufacturer’s instructions. Then, qPCR was performed using the 2^−∆∆CT^ and ACTB (β-actin) as a normalizer gene with Brilliant II SYBR^®^ Green QPCR Master Mix (#600828, Agilent Technologies, Santa Clara, CA, USA) following the manufacturer’s instructions. The qPCR reactions were performed with 250 nM of each primer (Table S3).

### 4.8. Adenosine Accumulation

The MKN-74 cell line and GCSCs were maintained under standard culture conditions (37 °C, 5% CO_2_) for 5 days. Then, GCSCs and non-GCSCs were washed with PBS 1X two times and incubated in 500 µL of Tyrode’s buffer (10 mM HEPES, 12 mM NaHCO_3_, 137 mM Choline Chloride, 2.7 mM KCl, 5 mM Glucose, and 1 mM CaCl_2_) for 1 h at 37 °C. Later, 200 µL of this incubation medium was mixed with 100 µL of citrate buffer (pH 4). Subsequently, the level of adenosine was determined using the Adenosine Quantification Assay Kit (MAK433-1KT, Sigma-Aldrich, Saint Louis, MO, USA) following the manufacturer’s recommendations. Adenosine concentrations (µM) were normalized to the total protein levels (µg). Extracellular total nucleoside in cells mediated by concentrative and equilibrative systems was also measured using Tyrode’s buffer containing sodium chloride instead of choline chloride. Sodium-dependent extracellular rates were obtained by subtracting adenosine extracellular in choline buffer from the total adenosine extracellular in buffer containing sodium chloride.

Subsequently, MKN-74 cells and GCSCs were maintained under standard culture conditions (37 °C, 5% CO_2_) for 5 days. Then, GCSCs were washed with PBS 1X two times and incubated in cell culture medium for 12 h at 37 °C. To assess the effect of blocking equilibrative nucleoside transporters on the accumulation of extracellular adenosine, NBTI was added to the cell culture medium for 1 h, at a concentration of 1 µM to inhibit ENT1 and 10 µM to inhibit ENT1 and ENT2 mediated transport, prepared in 0.1% DMSO. Subsequently, adenosine quantification proceeded as described above. Adenosine concentrations (nM) were normalized to total protein levels (μg).

### 4.9. CD73 and PAP Activity

The MKN-74 cell line and GCSCs were maintained under standard culture conditions (37 °C, 5% CO_2_) for 5 days. Then, they were exposed to 100 µM AMP (Cat#21094, Cayman Chemical Company, Ann Arbor, MI, USA) for 30 min in Tyrode buffer pH 6.0 supplemented with erythro-9-(2-hydroxy-3-nonyl) adenine (EHNA) (Cat#13352, Cayman Chemical Company, Ann Arbor, MI, USA), and with or without 10 uM PSB-12379 (Cat#28446, Cayman Chemical Company, Ann Arbor, MI, USA). The total incubation medium was immediately centrifuged at 4 °C for 5 min at 2500× *g*. The supernatant was kept on ice. After this procedure, 200 mL of supernatant was mixed with 100 mL of 0.1 M citrate phosphate buffer pH 4.0. Adenosine was quantified using the Adenosine Quantification Assay Kit (MAK433-1KT, Sigma-Aldrich), following the manufacturer’s recommendations. The values were expressed as the ratio between generated adenosine to total protein. CD73 activity was the fraction of AMPase activity inhibited by PSB-12379. PAP activity was the difference between total AMPase activity and the fraction of AMPase activity inhibited by PSB-12379.

### 4.10. Immunofluorescence and Image Analysis

Spheroids and MKN-74 adherent cells were fixed in 4% paraformaldehyde/0.1 M Na-phosphate buffer, pH 7.4 (PBS) for 30 min, washed several times with fresh PBS for 10 min, permeabilized with 0.1% Triton X-100 in PBS for 15 min, and incubated in 1% bovine serum albumin (BSA) in PBS/0.1% Triton X-100 for 30 min to block nonspecific binding. For staining, cells were incubated overnight at 4 °C with the following primary antibodies: anti-ENT1, anti-ENT2 (1:100 Santa Cruz Biotechnology, Santa Cruz, CA, USA), and anti-CD39, anti-CD73 (1:100 Cell Signaling Technology, Danvers, MA, USA). Each immunostaining was performed separately. After washing with PBS (three washes of 15 min each), cells were incubated with an Alexa Fluor 488-conjugated IgG secondary antibody (1:500; Invitrogen, Carlsbad, CA, USA) for 1 h at room temperature. Subsequently, additional washes were performed, and cell nuclei were stained with DAPI (1 μg/mL; Sigma-Aldrich, St. Louis, MO, USA) for 10 min at room temperature. Specifically, images were acquired using an Olympus BX41 fluorescence microscope equipped with a Euromex CCD camera (model 5.0 MP, DC12V/2A). Image acquisition and analysis were performed using the ImageFocus 4 software (Euromex Microscopes, The Netherlands). The objective lenses used for imaging were 20× and 40× magnification. For signal analysis, images were processed using ImageJ (NIH, Bethesda, Rockville, MD, USA), applying automated thresholding for segmentation and quantification of fluorescence intensity. The mean fluorescence intensity was measured in regions of interest (ROIs) defined in the plasma membrane and cytoplasm. Data were represented as mean ± SEM of three independent experiments. GraphPad 10.3.1 (GraphPad Software Inc., San Diego, CA, USA) was used for statistical analysis. *p*-values were calculated by the unpaired Student’s *t*-test comparison test. *p* < 0.05 was considered to be statistically significant.

### 4.11. Statistics

Data are presented as mean ± SEM from three independent experiments. Statistical analyses were performed using GraphPad version 10.3.1 (GraphPad Software Inc., San Diego, CA, USA). *p*-values were calculated using the non-parametric Mann–Whitney test for unpaired comparisons. A *p*-value < 0.05 was considered statistically significant.

## Data Availability

Data is contained within the article.

## Supplementary Materials

The following supporting information can be downloaded at https://www.mdpi.com/article/10.3390/ijms26157594/s1.

## Abbreviations

The following abbreviations are used in this manuscript:

GC	Gastric Cancer
CSCs	Cancer Stem Cells
GCSCs	Gastric Cancer Stem Cells
ATP	Adenosine Triphosphate
AMP	Adenosine Monophosphate
ENT	Equilibrative Nucleoside Transporter
CNT	Concentrative Nucleoside Transporter
