# Supplementary material for: Extracellular Adenosine in Gastric Cancer: The Role of GCSCs
Source: Int J Mol Sci. 2025 Aug 6;26(15):7594. doi: 10.3390/ijms26157594 (PMC12347490; doi:10.3390/ijms26157594)
Supplement: Supplementary file 1 [file ijms-26-07594-s001.zip › Densitometric Analysis - Western Blott Table S2.pdf]

Supplementary Table S2: Densitometric analysis of the western blotting experiments shown in Figure 2,4,7 and 8.

	Condition	Marker		β-actin	Ratio	Mean ± SEM	n	Relative amount (%)
**CD39**	Non GCSCs	7.998.740		18.119.054	0,44145461	0.426 ± 0.0111	4	100
		7.946.376		19.625.205	0,40490665			
		7.028.962		17.180.669	0,40912039			
		8.707.669		19.413.083	0,44854643			
	GCSCs	14.960.548		20.909.719	0,71548298	0.7119 ± 0.0092	4	167
		15.684.841		21.357.255	0,73440341			
		12.063.841		17.050.012	0,70755616			
		14.270.134		20.677.447	0,69013036			
**ENT1**	Non GCSCs	4.518.347		8.942.083	0,50529021	0.4481 ± 0.0312	4	100
		6.311.347		12.825.134	0,49210768			
		5.601.569		15.082.205	0,37140252			
		8.216.175		19.398.426	0,42354854			
	GCSCs	10.103.518		20.211.134	0,49989862	0.5269 ± 0.0256	5	118
		11.036.154		18.903.841	0,58380485			
		8.299.347		14.988.376	0,5537189			
		5.741.933		12.207.962	0,47034329			
		5.297.690		10.108.012	0,524108			
**ENT2**	Non GCSCs	8.941.125		19.406.134	0,46073705	0.4603 ± 0.0359	3	100
		8.500.539		21.357.255	0,39801646			
		9.894.296		18.943.891	0,52229481			
	GCSCs	11.583.660		14.826.669	0,78127191	0.7623 ± 0.0095	3	166
		9.128.246		12.154.426	0,75102238			
		7.478.004		9.910.134	0,75458152			
**CD73**	Non GCSCs	14.136.752		23.161.953	0,6103437	0.642 ± 0.0119	6	100
		18.054.217		27.724.711	0,65119586			
		18.630.510		27.523.347	0,67689842			
		17.363.045		28.568.054	0,60777836			
		19.524.045		29.181.832	0,66904795			
		17.554.731		27.563.225	0,63688959			
	GCSCs	15.342.217		25.260.146	0,6073685	0.6011 ± 0.0081	6	94
		15.417.752		26.468.761	0,58248862			
		15.715.681		26.877.518	0,58471474			
		17.819.338		28.424.933	0,62689112			
		15.831.217		26.207.054	0,60408228			
		12.256.217		22.796.761	0,53762975			
