# Supplementary material for: Extracellular Adenosine in Gastric Cancer: The Role of GCSCs
Source: Int J Mol Sci. 2025 Aug 6;26(15):7594. doi: 10.3390/ijms26157594 (PMC12347490; doi:10.3390/ijms26157594)
Supplement: Supplementary file 1 [file ijms-26-07594-s001.zip › Primary Antibody Table S1.pdf]

Table S1. List of antibodies used in this study.

Target	Antibody	Company
ENT1	sc-377283	Santa Cruz Biotechnology
ENT2	sc-373871	Santa Cruz Biotechnology
CD73	D7F9A	Cell Signaling
PAP	RD.MAB6240	R&D Systems
CD39	E5A6L	Cell Signaling
β-actin	sc-47778	Santa Cruz Biotechnology
